# Updated COVID‐19 clearance time among patients with cancer in the Delta and Omicron waves

**DOI:** 10.1002/cam4.6311

**Published:** 2023-07-01

**Authors:** Zachary M. Avigan, Rodrigo Paredes, Leora S. Boussi, Barbara D. Lam, Meghan E. Shea, Matthew J. Weinstock, Mary Linton B. Peters

**Affiliations:** ^1^ Department of Medicine Beth Israel Deaconess Medical Center Boston Massachusetts USA; ^2^ Division of Hematology/Oncology, Department of Medicine Beth Israel Deaconess Medical Center Boston Massachusetts USA

**Keywords:** COVID clearance, COVID‐19

## Abstract

**Background:**

COVID‐19 infection delays therapy and in‐person evaluation for oncology patients, but clinic clearance criteria are not clearly defined.

**Methods:**

We conducted a retrospective review of oncology patients with COVID‐19 at a tertiary care center during the Delta and Omicron waves and compared clearance strategies.

**Results:**

Median clearance by two consecutive negative tests was 32.0 days (Interquartile Range [IQR] 22.0–42.5, *n* = 153) and was prolonged in hematologic malignancy versus solid tumors (35.0 days for hematologic malignancy, 27.5 days for solid tumors, *p* = 0.01) and in patients receiving B‐cell depletion versus other therapies. Median clearance by single negative test was reduced to 23.0 days (IQR 16.0–33.0), with recurrent positive rate 25.4% in hematologic malignancy versus 10.6% in solid tumors (*p* = 0.02). Clearance by a predefined waiting period required 41 days until an 80% negative rate.

**Conclusions:**

COVID‐19 clearance remains prolonged in oncology patients. Single‐negative test clearance can balance delays in care with risk of infection in patients with solid tumors.

## Abstract 2

We conducted a retrospective review of oncology patients with COVID‐19 infection at a single tertiary care center during the Delta and Omicron waves and compared clearance strategies. Median clearance as defined by two consecutive negative tests was 32 days, and was prolonged in hematologic malignancy and in patients who received B‐cell depletion. Single‐negative test clearance showed high recurrence in patients with hematologic malignancy but may be appropriate for patients with solid tumors.

## 1 BACKGROUND

The COVID‐19 pandemic has presented a particular challenge in the care of patients with malignancy, as they are at higher risk for severe disease,^1^, ^2^, ^3^, ^4^ prolonged viral shedding,^5^, ^6^, ^7^ and diminished humoral response to vaccination^8^, ^9^, ^10^ that is further exacerbated by immunosuppressive cancer‐directed therapies.^11^, ^12^ COVID infection frequently leads to delays in care and in‐person clinic visits to protect against worsening disease and prevent transmission to other vulnerable patients, and both the American Society of Clinical Oncology (ASCO) and the UK National Institute for Health and Care Excellence (UK‐NICE) recommend pausing or delaying treatment during active infection.^13^, ^14^ However, there are no clear guidelines for determining clearance and when patients can safely return to clinic.

Our group previously published a report of prolonged COVID‐clearance times in oncology patients.^7^ Based on these data and Centers for Disease Control and Prevention (CDC) isolation guidelines for patients with moderate to severe immune compromise,^15^ during the time of this study from October 2021 to March 2022, our center required two consecutive negative PCR tests prior to return to clinic. However, with rising vaccination rates and evolving viral variants, it is unclear whether this remained the optimal strategy for patients with different cancer subtypes or treatment modalities, and criteria for clearance and resumption of care should be revisited. In this study, we performed an updated analysis of viral clearance during the initial peaks of the Delta and Omicron waves at our center to assess the validity of this clearance approach.

## 2 METHODS

We identified patients at our academic medical center with active malignancy who tested positive for COVID‐19 by PCR testing between October 1, 2021 and March 31, 2022 and had at least one subsequent negative assay. During this period, PCR testing at our center was performed using one of the following four assays approved under Emergency Use Authorization by the US Food and Drug Administration: Abbott RealTime SARS‐CoV‐2 assay (Abbott Laboratories), Alinity m SARS‐CoV‐2 assay (Abbott Laboratories), Abbott ID NOW COVID‐19 assay (Abbott Diagnostics Scarborough, Inc.,), and Xpert Xpress SARS‐CoV‐2 test (Cepheid).

Chart review was performed under an IRB‐approved protocol (Protocol: 2022P000110) for patient demographics, comorbidities, cancer‐directed therapy, COVID vaccination status, and all COVID test results during the study period; COVID variant was inferred from the time of infection based on the dominant strain in the region. Active treatment was recorded at the time of initial infection and was defined as chemotherapy, targeted therapy (e.g., targeted monoclonal antibodies, tyrosine kinase inhibitors), or immunotherapy (e.g., checkpoint inhibitors) within 3 months; B‐cell depletion (e.g., rituximab) within 6 months; or transplant or adoptive cell therapy (e.g., CAR T‐cell therapy) within 1 year, based on prior data showing increased risk of severe infection and blunted immune response to vaccination at these intervals after each therapy.^16^, ^17^, ^18^, ^19^, ^20^, ^21^, ^22^ Patients were defined as fully vaccinated if they received at least two mRNA‐based vaccines (Pfizer or Moderna) or at least one adenovirus vaccine (Johnson & Johnson/Janssen), boosted if they received a full series plus at least one additional booster vaccine, and partially vaccinated if they received only one mRNA vaccine.

COVID clearance time was defined as the number of days from initial infection until two consecutive negative PCR tests. Subgroups were compared using the Mann–Whitney *U* test for two groups and the Kruskal–Wallis test for greater than two groups, followed by pairwise Mann–Whitney *U* testing if significant. Rates of repeat positive test after initial negative were compared by Fisher's exact test, and comparison of negative rates over time was performed by Kaplan–Meier analysis with log‐rank (Mantel–Cox) test for significance. All statistical analyses were performed in GraphPad Prism version 9.5.0 (Dotmatics).

## 3 RESULTS

169 patients met inclusion criteria of which 153 patients had documented clearance defined by two consecutive negative tests, with demographic data shown in Table 1. The average age was 60.6 years, and a majority of patients identified as female (57.5%) and white (63.4%). The most common comorbidity was diabetes (17.0%), followed by COPD or other severe lung disease (8.5%). Patients had primarily solid tumors (61.4%), and 79.7% were on active therapy. 75.8% of patients were fully vaccinated against COVID‐19, including 24.2% who had received at least one booster vaccine, and 83.0% had at least partial vaccination.

**TABLE 1:** Patient demographic data.

Demographic	Number (%)
Age (mean ± SD)	60.6 ± 14.6
Gender	
Male	65 (42.5%)
Female	88 (57.5%)
Race/ethnicity	
White	97 (63.4%)
Black or African American	33 (21.6%)
Hispanic	27 (17.6%)
Other	25 (16.3%)
Comorbidities/risk factors	
COPD	7 (4.6%)
Other severe lung disease	6 (3.9%)
CHF	11 (7.2%)
Diabetes	26 (17.0%)
Active smoking	8 (5.2%)
Autoimmune disease	4 (2.6%)
HIV	0 (0%)

Abbreviations: COPD, chronic obstructive pulmonary disease; CHF, congestive heart failure; HIV, human immunodeficiency virus.

The median clearance time in the cohort was 32.0 days (Interquartile Range [IQR] 22.0–42.5), with subgroups shown in Table 2. The clearance time was significantly faster in solid tumors versus hematologic malignancy (27.5 vs. 35.0 days, *p* = 0.01). Additionally, there was a significant difference in clearance time by treatment type (*p* = 0.002). Specifically, there was a significant increase in clearance time in patients treated with B‐cell depletion (55.5 days) versus chemotherapy or targeted therapy alone (31.0 days, *p* = 0.004), immunotherapy (24.0 days, *p* < 0.0001), or transplant/adoptive cell therapy (35.0 days, *p* = 0.04). There was no significant difference by vaccination status or between Delta and Omicron waves.

**TABLE 2:** Median COVID clearance times by subgroup. IQR, interquartile range.

Subgroup	Number (%)	Clearance time, days (IQR)	*p*‐Value
Malignancy			**0.01**
Solid	94 (61.4%)	27.5 (19.0–38.25)	‐
Hematologic	59 (38.6%)	35.0 (24.0–51.0)	‐
Treatment			0.25
Active treatment	122 (79.7%)	32.5 (22.75–45.0)	‐
No active treatment	31 (20.3%)	27.0 (19.0–37.0)	‐
Treatment Type			**0.002**
Chemotherapy/targeted therapy alone	61 (50.0%)	31.0 (23.0–44.0)	**0.004** ^a^
B‐cell depletion	6 (4.9%)	55.5 (47.25–66.0)	‐
Immunotherapy	34 (27.9%)	24.0 (17.75–38.0)	**<0.0001** ^a^
Stem cell/adoptive cell transplant	13 (10.7%)	35.0 (27.0–54.0)	**0.04** ^a^
Vaccination			0.91
Full series with booster	37 (24.2%)	32.0 (21.0–40.0)	
Full series only	79 (51.6%)	31.0 (22.0–49.0)	‐
Partial	11 (7.2%)	33.0 (18.0–60.0)	‐
None	26 (17.0%)	31.5 (22.75–41.0)	‐
COVID variant			0.69
Delta	39 (25.5%)	32.0 (22.0–45.0)	‐
Omicron	114 (74.5%)	31.5 (21.75–42.25)	‐

*p*‐values <0.05 were bolded.

^a^ Compared to B‐cell depletion.

If clearance was instead defined as a single negative PCR test, median clearance time was reduced from 32.0 to 23.0 days (IQR 16.0–33.0), and there again was a significant difference in patients with hematologic versus solid tumors and in those treated with B‐cell depletion versus other therapies. However, 16.3% of patients (25/153) had a subsequent positive result after the first negative, which was particularly enriched in patients with hematologic malignancy as opposed to those with solid tumors (25.4% vs. 10.6%, *p* = 0.02), as well as in patients receiving transplant/adoptive cell therapy (46.2%).

**FIGURE 1:**
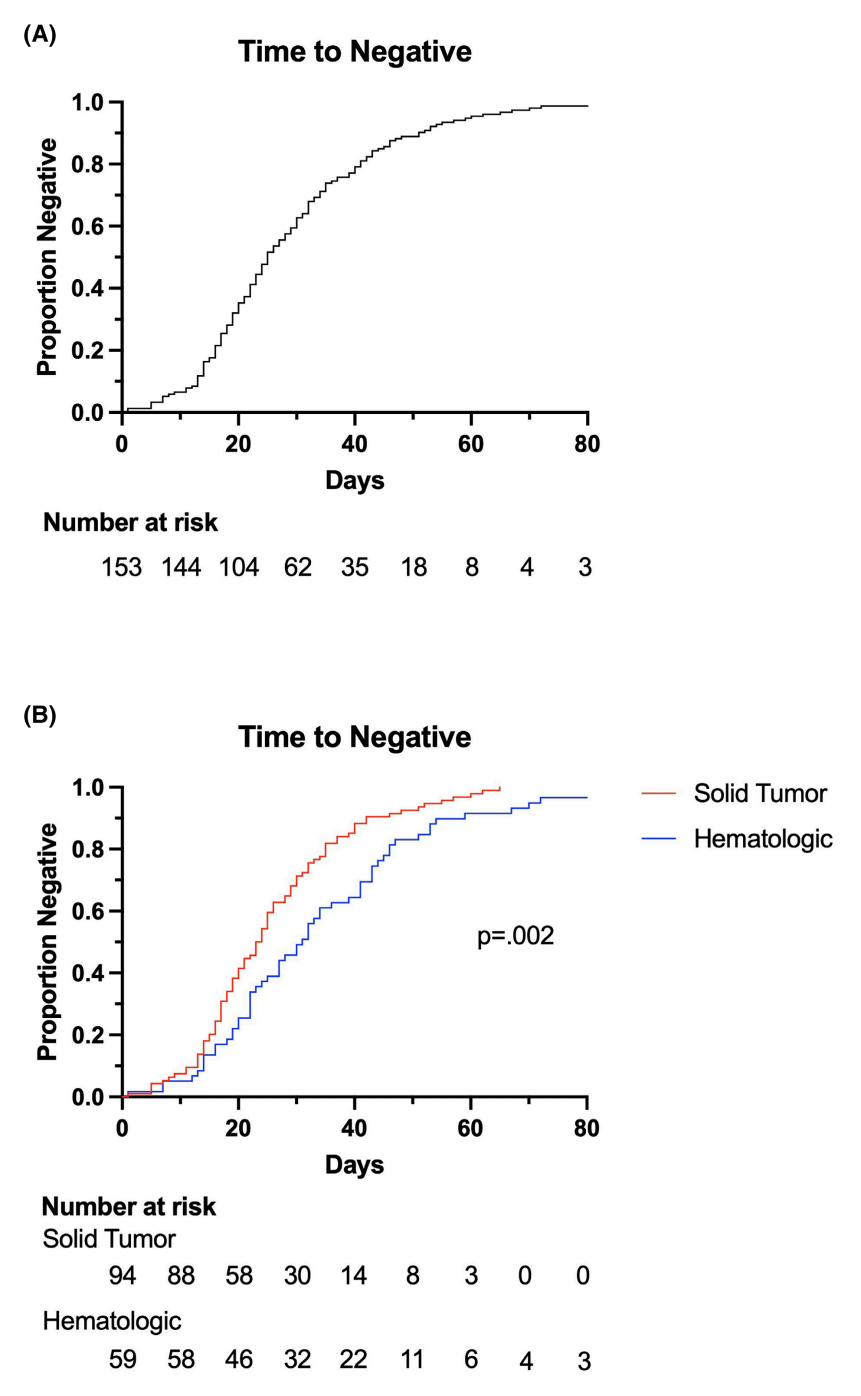
Proportion of COVID‐negative patients with number at risk across time in the overall cohort (A) and comparing solid and hematologic malignancies (B).

We next examined a clearance approach with a predefined waiting period by assessing what proportion of patients had a durable negative result at different timepoints (Figure 1). Of note, it took 41 days to reach a true negative rate of 80% and 60 days to reach a true negative rate of 95% in the overall cohort (Figure 1A). While the solid tumor population again cleared faster than patients with hematologic malignancy (Figure 1B, *p* = 0.002), an 80% negative rate was not reached in the solid tumor cohort until 35 days.

## 4 DISCUSSION

The COVID‐19 pandemic has continued to restrict access to medical care and limit in‐person evaluation, with an undue burden on patients with malignancy and other immune compromise.^23^, ^24^, ^25^, ^26^ Current guidelines recommend delaying cancer‐directed therapy to prevent worsening infection,^13^, ^14^ but clearance criteria are not clearly defined. The CDC's current recommendations for isolation of immunocompromised patients remains 20 days in addition to two negative PCR tests,^15^ but with increasing vaccination rates and evolving viral variants, it is not known whether this should continue to be universally applied to oncologic care.

In this study, we present an updated analysis of COVID‐19 clearance times in patients with malignancy using a two‐negative‐test criterion. Despite high vaccination rates, our center's oncology patients continue to have longer clearance times than reported in the general population,^27^, ^28^ with a median clearance time of 32 days. Of note, we showed that clearance times were significantly longer in patients with hematologic malignancy and those receiving B‐cell depletion, consistent with prior studies.^4^, ^6^, ^29^ These data suggest the possibility of different clearance protocols for varying disease types or degrees of immunosuppression.

Using a single negative test for clearance would decrease burden on patients and healthcare resources and could expedite care. However, the greatest concern is ongoing COVID positivity, either due to a false negative test or residual low level viral shedding, which may be worsened by immunosuppressive therapy. As expected, a single‐negative approach reduced clearance time from 32 to 23 days. There was a 16.3% recurrence rate, but recurrence was significantly lower in patients with solid versus hematologic malignancies after a first negative test. Alternatively, adopting a predefined waiting period for return to clinic could similarly reduce costs and patient coordination associated with repeat testing; unfortunately, based on the heterogeneity of clearance times in this cohort, selecting the appropriate number of days to ensure a safe COVID‐negative rate may actually increase waiting times for many patients. Based on these data, it is likely reasonable to use a single‐negative test strategy in some subgroups, and our center and others have adopted this policy for patients with solid tumors to advance care while balancing risk of ongoing infection.

There was no association in this cohort between vaccination status and clearance time, and previous analyses have shown increased breakthrough infections and limited humoral response to vaccines in some oncology patients, particularly those with hematologic malignancy.^8^, ^9^, ^10^, ^30^, ^31^ However, there are multiple reasons for caution in interpreting these results. This study evaluated clearance time but did not assess severity of illness, and there is substantial data in the literature showing the efficacy of vaccination in reducing morbidity and mortality in immunocompromised patients with cancer and those with malignancy.^32^, ^33^, ^34^ Additionally, given the study period evaluated, less than one third of vaccinated patients in this cohort received booster vaccinations, and prior studies have shown decreased protection without booster vaccines in this population.^31^, ^32^, ^34^, ^35^ Finally, we are fortunate to have high vaccination rates at our cancer center, and only a small number of unvaccinated patients were included in this study, which reduced the power of this study to assess the role of vaccination and limits generalizability to unvaccinated populations.

These data were evaluated to better inform cancer center‐wide policies regarding safe return to oncology clinic; therefore, a binary, hospital‐based PCR assay was used for patient assessment to create a clearance algorithm and optimize patient safety. However, it should be noted that prolonged PCR‐positivity alone is an imprecise biomarker for risk of severe illness, chemotherapy administration, or COVID‐19 transmissibility that should preclude exposure to other patients in clinic, particularly with low viral load or increased cycle threshold.^36^, ^37^ Cancer‐directed therapy has been safely used in some patients with active COVID‐19 infection and should be considered on an individual basis.^38^, ^39^ At our center, patients requiring therapy who remain COVID‐positive for >10 days are reviewed by a multidisciplinary panel to assess safety of treatment at a separate infusion site while awaiting clearance for return to clinic. Similar individualized approaches to oncologic therapy are likely warranted in patients with prolonged PCR positivity.

Further limitations of this study include less frequent testing at the studied stage of the pandemic, which may artificially inflate documented clearance times. While immunosuppressed patients requiring close monitoring are more likely to be tested, stable patients using at‐home tests or with shorter duration of symptoms may be underrepresented in this cohort. Home antigen‐based testing was not directly assessed as a marker of clearance in this study, as it is not routinely used in our cancer center. While COVID‐19 antigen tests have been shown to be less sensitive than PCR‐based testing,^40^, ^41^, ^42^ home testing may represent an additional avenue to reduce delays in return to clinic for patients who are asymptomatic or clinically improving. Additionally, it was difficult to track the effects of outpatient oral COVID‐directed therapies (e.g., nirmatrelvir/ritonavir) on clearance time, as they may have been prescribed by other non‐oncologic providers, though it is likely that the majority of patients in the study received treatment given the predominant practice during the study period. Finally, these results should not be generalized to patients who are not undergoing active oncologic care.

## 5 CONCLUSIONS

COVID‐19 infection remains a significant barrier to safe treatment for malignancy, but there are no clear guidelines for return to clinic or resumption of therapy. In this single‐center study, we showed ongoing delays in COVID‐19 clearance in cancer patients, particularly in patients with hematologic malignancy. While clearance times remain prolonged, this study highlights the possibility of more aggressive clearance strategies in some subgroups, such as single‐negative testing in patients with solid tumors, to help progress oncologic care during the pandemic.

## AUTHOR CONTRIBUTIONS

**Zachary M Avigan:** Conceptualization (equal); data curation (lead); formal analysis (lead); methodology (equal); writing – original draft (lead); writing – review and editing (lead). **Rodrigo Paredes:** Data curation (equal). **Leora S Boussi:** Data curation (equal). **Barbara D Lam:** Data curation (equal); writing – review and editing (equal). **Meghan E Shea:** Supervision (equal); writing – review and editing (equal). **Matthew J Weinstock:** Supervision (equal). **Mary Linton B Peters:** Conceptualization (lead); methodology (lead); resources (lead); supervision (lead); writing – review and editing (equal).

## CONFLICT OF INTEREST STATEMENT

MES: Reports advisory role for GSK as well as institutional research funding from Clovis Oncology, Bristol‐Myers Squibb, Tesaro/GSK, AstraZeneca, and Lilly, all outside the submitted work. MLBP: Discloses research funding from NCI (K08CA248473), as well as institutional research funding from Taiho, AstraZeneca, Exelixis, BeiGene, Berg Pharma, Merck, Bayer, NuCana, Lilly, and Helsinn Therapeutics, all outside the submitted work. Other authors have no potential conflicts.

## ETHICS STATEMENT

Patient data was reviewed and collected under an IRB‐approved protocol (Protocol: 2022P000110).

## Data Availability

The data that support the findings of this study are available on request from the corresponding author. The data are not publicly available due to privacy or ethical restrictions.
